# Development of an Acid-Resistant *Salmonella* Typhi Ty21a Attenuated Vector For Improved Oral Vaccine Delivery

**DOI:** 10.1371/journal.pone.0163511

**Published:** 2016-09-27

**Authors:** Madushini N. Dharmasena, Catherine M. Feuille, Carly Elizabeth C. Starke, Arvind A. Bhagwat, Scott Stibitz, Dennis J. Kopecko

**Affiliations:** 1 Laboratory of Mucosal Pathogens and Cellular Immunology, Food and Drug Administration-Center for Biologics Evaluation and Research, New Hampshire Avenue, Silver Spring, Maryland, United States of America; 2 Environmental Microbial and Food Safety Laboratory, Agricultural Research Service, United States Department of Agriculture, Beltsville, Maryland, United States of America; University of Helsinki, FINLAND

## Abstract

The licensed oral, live-attenuated bacterial vaccine for typhoid fever, *Salmonella enterica* serovar Typhi strain Ty21a, has also been utilized as a vaccine delivery platform for expression of diverse foreign antigens that stimulate protection against shigellosis, anthrax, plague, or human papilloma virus. However, Ty21a is acid-labile and, for effective oral immunization, stomach acidity has to be either neutralized with buffer or by-passed with Ty21a in an enteric-coated capsule (ECC). Several studies have shown that efficacy is reduced when Ty21a is administered in an ECC versus as a buffered liquid formulation, the former limiting exposure to GI tract lymphoid tissues. However, the ECC was selected as a more practical delivery format for both packaging/shipping and vaccine administration ease. We have sought to increase Ty21a acid-resistance to allow for removal from the ECC and immune enhancement. To improve Ty21a acid-resistance, glutamate-dependent acid resistance genes (GAD; responsible for *Shigella spp*. survival at very low pH) were cloned on a multi-copy plasmid (pGad) under a controllable arabinose-inducible promoter. pGad enhanced acid survival of Ty21a by 5 logs after 3 hours at pH 2.5, when cells were pre-grown in arabinose and under conditions that promote an acid-tolerance response (ATR). For genetically 100% stable expression, we inserted the *gad* genes into the Ty21a chromosome, using a method that allowed for subsequent removal of a selectable antibiotic resistance marker. Further, both bacterial growth curves and survival assays in cultured human monocytes/macrophages suggest that neither the genetic methods employed nor the resulting acid-resistance conferred by expression of the Gad proteins in Ty21a had any effect on the existing attenuation of this vaccine strain.

## Introduction

**Fig 1:**
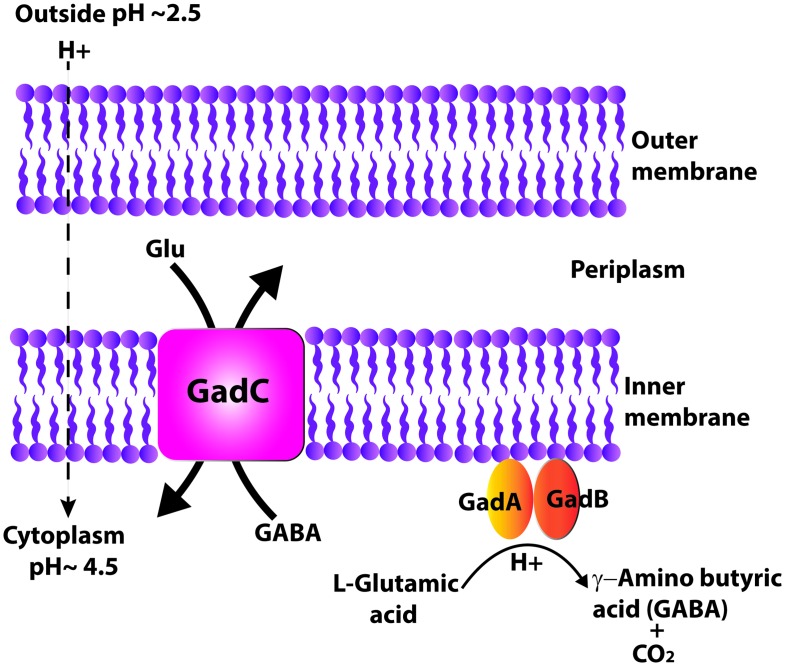
Schematic diagram of GDAR system (adapted from Zhao et al., 2010). This system consists of paralogous GadA and GadB decarboxylases and an inner-membrane antiporter GadC. GadA and GadB are pyridoxal 5’-phosphate (PLP)- dependent enzymes that convert L-glutamic acid into GABA and CO_2_, in a reaction that consumes a cytoplasmic proton. The inner membrane antiporter GadC transports GABA out of the cell in exchange for more glutamate.

The licensed oral live, attenuated bacterial vaccine for typhoid fever consists of the *galE*, Vi-capsule negative *Salmonella enterica* serovar Typhi strain Ty21a. This vaccine has been administered to more than 200 million recipients worldwide over 25 years and has never reverted to virulence [1, 2]. In addition, there has never been a documented report of post-vaccination, Ty21a-related inflammatory arthritis (i.e., Reiter's syndrome), which can occur following natural infections with *Shigella*, *Yersinia*, *Campylobacter*, and nontyphoid serovars of *Salmonella* and therefore potentially following oral administration of attenuated live vaccines containing these organisms. Ty21a also affords long-term protection (> 8 years) after 3 doses given over a short 5 day immunization period [3]. For these reasons, we and others have utilized this bacterial strain as a versatile oral vaccine vector for expression of diverse foreign antigens [4]. For example, Ty21a has already been engineered to stably express a variety of targeted LPS and protein antigens that protect against shigellosis [5–7], anthrax [8], plague (M. R. Foote, M. N. Dharmasena, T. T. Wai, M. Osorio and Kopecko, unpublished data), and human papilloma virus in both animal models [9, 10] and human challenge studies [11]. Ty21a induces mucosal, humoral, and cellular immune responses and may be utilized as a multivalent combination vaccine vector that is inexpensive to produce [12]. The ability of enteric bacteria, after ingestion, to withstand acidic stresses in the stomach is crucial for subsequent successful colonization in the gastrointestinal tract. Each microorganism contains different combinations of acid tolerance response (ATR) systems and/or acid resistance (AR) systems and, thus, exhibits a different degree of resistance to acid-stress [13]. The AR systems typically protect stationary-phase cells from an extreme acid stress without pre-adaptation. Five bacterial AR pathways, AR1-AR5, have been described previously [14]. AR1 is found in *Escherichia coli* and is poorly characterized. The AR2-AR5 systems generally consist of a specific decarboxylase enzyme that is induced at low pH and utilizes excess protons to decarboxylate a specific amino acid (e.g. aspartic acid, phenylalanime, lysine, or glutamic acid), and an antiporter that transports the decarboxylated product extracellularly (as depicted in Fig 1) [14]. The ability of *E*. *coli*, *Shigella spp*., *Listeria monocytogenes* and *Lactococcus lactis* to withstand extreme acidic pH (below pH 2.5) primarily relies on the most potent AR system, AR2, also known as the GDAR (glutamate-dependent acid resistance) pathway. This system consists of a glutamate γ-aminobutyric acid (GABA) antiporter, GadC, and two glutamate decarboxylase isozymes encoded by the paralogous *gadA* and *gadB* genes [15]. AR3, found in *E*. *coli* and *Salmonella spp*., is an arginine-dependent acid resistance system that provides modest protection at pH 2 to 4 [16]. AR4, the main AR found in *Vibrio cholerae*, is lysine-dependent and functions in moderately acidic conditions around pH 5. AR5 consists of an inducible decarboxylase that has not been well studied to date [14].

ATR systems, in contrast, comprise moderate acid-stress responses that result in the induction of a set of proteins called acid shock proteins (ASPs), that are typically induced at mildly acidic pH, and that prepare the cell for limited acidic pH exposure. ATRs have been mostly described in *Salmonella enterica* serovar Typhimurium. The ASPs appear to act by repairing, degrading and preventing formation of damaged macromolecules caused by acid stress. For example, the ASPs include DNA repair enzymes *polA* and *ada*, which repair acid-damaged DNA. The ATR also shifts the fatty acid profile of the cell, contributing to increased hydrophobicity and thus decreasing proton influx across the membrane. Key proteins that regulate subsets of ASPs include: the alternative sigma factor RpoS; the iron uptake regulator, Fur; and two component regulatory systems such as PhoPQ and OmpR/EnvZ. [13, 17–19]. Note that the *S*. Typhi parent strain Ty2 and live, attenuated *S*. Typhi vaccine candidates, such as Ty21a, derived from the Ty2 parent are *rpoS* mutants [20]. However, Ty21a is more acid-sensitive than Ty2. Apparently, yet uncharacterized mutation(s) introduced by random mutagenesis during virulence attenuation (Germanier & Furer, 1975) have further reduced Ty21a’s ability to survive at low pH. In fact, Ty21a contains a large number of single nucleotide polymorphisms (SNPs) apparently introduced during random mutagenesis [21–23], one or more of which may affect ATRs.

Although *Salmonella* encode inducible ATR, AR3, AR4 and AR5 systems, this genus is only moderately acid-resistant, possibly due to absence of the extreme acid-resistance pathway AR2, the GDAR pathway [13, 24]. An infectious dose of *Salmonella* Typhi is estimated at 10^3^ to 10^6^ CFU [25, 26], whereas *Shigella* species require a much smaller inoculum of 10 to 500 CFU to cause dysentery [27]; and it has been suggested that infectious dose correlates strongly with the level of acid resistance of enteric pathogens [13, 25, 27].

As a vaccine, Ty21a has sub-optimal acid-lability; hence stomach acidity has to be neutralized or bypassed as Ty21a is administered orally. In early clinical trials, the stomach acidity of the volunteers was neutralized with bicarbonate buffer prior to ingestion of Ty21a in a reconstituted liquid formulation [28]. For practical reasons, however, Ty21a was ultimately formulated into ECC to bypass gastric acid exposure and is administered before meals or 2 hours after a meal [29]. However, reports indicate that ECC delivery of Ty21a results in moderately reduced immunogenicity versus the buffered liquid delivery format [28]. Interestingly, this reduced immunogenicity of ECC-delivered Ty21a may result partially from the lack of subneutral pH exposure, an important signal that prepares enteric bacteria for stresses of the intestine and bacterial-host cell interactions. Furthermore, the ECC physically limits exposure of the vaccine strain to only the lower small intestine/large intestine, which likely limits the overall immune response. Thus, the encapsulated form of Ty21a engenders a moderate level of immunity [30, 31]. In fact, the liquid oral formulation of Ty21a was found to be superior to ECC in field trials, possibly due to the fact that bacteria in liquid formulation have access to the tonsils and other lymphoid tissues in the oral cavity and throughout the gastrointestinal tract and, therefore elicit an enhanced immune response [29]. Thus, if the Ty21a vaccine vector can be engineered to survive the low pH of the stomach for 2–3 hours (i.e. normal transit time through a full stomach), this modification may allow for a final Ty21a delivery format as a rapidly dissolvable wafer without the need for buffer. This mode of delivery is more attractive than the current large bullet-sized ECC (which children have difficulty swallowing) or a 200 ml liquid formulation (impractical for shipping because of weight). Furthermore, a dissolvable wafer format may allow for active immunization in the oral cavity as well as throughout the gastrointestinal tract, which could enhance both the overall immune response and vaccine uptake by young children. Additionally, improving acid resistance should increase the number of viable cells that reach the small intestine and may therefore reduce both the cfu/dose and dose number required for long-lasting, high level protection.

In this study, we have demonstrated that the survival of the original (acid-sensitive) vaccine strain Ty21a can be significantly enhanced (10^5^-fold) by expressing cloned *Shigella gad* genes from an inducible promoter. We also determined growth conditions necessary for optimal acid survival, including both ATR and AR systems, of this acid-resistant Ty21a. These improvements may enhance the value of Ty21a as an oral typhoid vaccine and as a versatile vaccine vector for construction of single purpose and multi-agent vaccines against numerous infectious diseases and bioterrorism agents.

## Material and Methods

### Bacterial strains, plasmids, and growth conditions

The bacterial strains and plasmids utilized herein are described in Table 1. Ty21a was obtained from a fresh Vivotif ^™^ (Crucell) capsule, grown aseptically and stored as a limited passage genetic seed stock. All bacteria were grown in Difco tryptic soy broth (TSB) or tryptic soy agar (TSA). Culture conditions used in this study are, TSB at pH 4.5 or 5.5 buffered with 2-(4-morpholino) ethane sulfonic acid (MES, Fisher, NY), TSB at pH 6.5 or 7.5 buffered with 3-(4-Morpholino) propane sulfonic acid (MOPS, Fisher, PA) or commercial TSB (pH 7.2). All standard TSB medium contains 0.25% glucose unless mentioned otherwise (Difco). To examine the effect of glucose on expression of bacterial acid-resistance, TSB media lacking glucose was also employed. Cultures were supplemented with 0.01% galactose, 0.75% arabinose, and 1 mM glutamate, unless specified otherwise. All cultures were grown aerobically in a shaking incubator at 200 rpm and at 37°C, unless noted otherwise. Anaerobic cultures were grown standing at 37°C in a BBL anaerobic jar using an anaerobic Gaspak E3 (BD, NJ). For stationary phase cultures, a single colony from a fresh TSA plate was inoculated into 10 ml TSB and grown for either 18 or 24 hours. To obtain log phase cultures, overnight cultures were diluted 1:100 and grown until OD_600_ = 0.4–0.5. Plasmid-containing strains were selected in appropriate growth medium containing ampicillin (Amp; 100 μg/ml), chloramphenicol (Cam; 35μg/ml), or kanamycin (Kan; 30 μg/ml). To support the growth of *E*. *coli asd* mutants, TSB and TSA media was supplemented with 400μg /ml diaminopimelic acid (DAP, Sigma, MO). For growth comparisons, Ty21a and derivative strains were grown at 37°C in TSB with 0.01% galactose and 1 mM glutamate supplementation and either with or without 0.75% arabinose, using a BioTek Cytation 3 imaging reader with shaking incubator at 200 rpm and automatic OD_600_ readouts. All constructed plasmids and chromosomal integrants (i.e. PCR products originating from the site of integration) were sequenced and the sequences were assembled and analyzed using Vector NTI suite 9.0 software (Invitrogen). Sequences are available under GENBANK accession numbers KJ870102 for the *tviD-vexA* insert region of Ty21a-Gad, KJ870100 for pGad, KJ870099 for pMD.SD, KJ870098 for pMD.SV and KJ870101 for pMD.SD.Gad.

**Table 1:** Bacterial strains and plasmids.

Strain or plasmid	Genotype or description	Reference or source
Strains		
*E*. *coli* DH5α λpir	*supE44*, *hsdR17*, *recA1*, *endA1*, *gyrA96*, *thi-1*, *relA1*, *λpir*	Susan Gottesman lab
*E*. *coli* RHO3 (Dap-)	SM10 λpir, Kan^S^, *Δasd*::*FRT*, *ΔaphA*::*FRT*	[33]
*S*. *enterica* serovar Typhi Ty2	Wild type strain, *rpoS*	Kopecko lab
*S*. *enterica* serovar Typhi Ty21a	*galE*, *ilvD*, *viaB (Vi-*,*) H*_*2*_*S-*, *rpoS*	[23]
Ty21a *vexA* merodiploid (MD290)	Two copies of *vexA* and FRT site	This study
Ty21a-Gad (MD297)	*S*. *flexneri gad* genes integrated into *tviD*-v*exA* on the chromosome, Kan^s^, Amp^s^, Cam^s^	This study
*S*. *flexneri* 2457T CFS100	Avirulent isolate deleted for the invasion plasmid	Shelly Payne lab
Plasmids		
pCP20	yeast Flp recombinase gene FLP, Cm^r^, Amp^r^, ts-rep^a^	[34]
pMD-SV	Suicide vector with R6K, RP4 and *vexA*	This study
pMD-SD	Suicide vector with R6K, RP4 and *tviD*	This study
pMD-SD-gad	*gadABC* cloned into pMD-SD	This study
pNW129	P15A rep, Kan^r^	[37]
pGad	*gadABC* cloned into pNW129	This study

^a^ ts-rep; temperature-sensitive replication.

### Acid resistance assay

Five hundred microliters of TSB-pregrown bacterial cultures were directly diluted into 10 ml of TSB-MES pH 2.5. Following incubation at 37°C, samples were retrieved at time 0, 1, 2 and 3 hours and appropriate dilutions in TSB were immediately plated onto TSA plates to determine viable count. The lowest level of detection is 1 CFU in 100 μl of undiluted sample. If no CFU were found when 100 μl of the undiluted samples were plated, the result was considered to be 1 CFU for calculation purposes. The % survival (input to output ratio) is presented as mean ± SEM from three or more independent experiments. Statistical analyses were performed by means of an unpaired *t* test using GraphPad Prism version 5. A *p* value of <0.05 (two tailed) was considered to be statistically significant (**).

### Construction of acid resistant strains

Standard molecular biology techniques were used for cloning. The restriction endonucleases and ligase were purchased from New England Biolabs (NEB, MA) or Fermentas, and Phusion polymerase (Fisher) was used for all PCRs. Plasmids pGad and pMD-SD were synthesized by GenTech, CA. pMD-SD contains an R6K origin of replication, RP4 mob site [32], Kan^R^ cassette, FRT sites (flippase recognition target) and part of the *S*. Typhi *tviD* gene. In order to construct pMD-SV, the *vexA* gene (~ 1000bp) was PCR-amplified with primers prMND205.VexA.F and prMND206.VexA.R (Table 2) from Ty21a genomic DNA, digested with *Bam*HI and *Nde*I and ligated to pMD-SD that was digested with the same restriction enzymes and gel-purified (to eliminate the *tviD* gene of pMD-SD). In order to construct pMD-SD-Gad, the *gad* genes with arabinose promoter (~6500bp) were PCR amplified with primers prMND214.Gad.F.*Xho*I and prMND215.Gad.R.*Kpn*I from plasmid pGad, digested with *Xho*I and *Kpn*I and ligated to pMD-SD that was digested with the same restriction enzymes. Ty21a *vexA* merodiploid strain MD290 and Ty21a-Gad strain MD 297 were constructed as shown below. *E*. *coli* donor strain RHO3 [33] carrying pMD-SV was conjugated with recipient strain Ty21a on a DAP-supplemented TSA plate and selection for kanamycin-resistant Ty21a transconjugants was imposed by plating on TSA plates with kanamycin. Kanamycin-resistant Ty21a transconjugants were transformed with pCP20 [34], and Cm^r^ transformants were selected at 30°C, after which a few isolates were non-selectively colony-purified at 37°C and then tested for loss of all antibiotic resistances as described previously [7] to construct Ty21a *vexA* merodiploid (strain MD290). Ty21a *vexA* merodiploid was conjugated with RHO3 containing pMD-SD, selection was imposed for kanamycin-resistant transconjugants and those were transformed with pCP20 as before to construct Ty21a-Gad (strain MD 297). Ty21a-Gad was analyzed by PCR to confirm chromosomal integration, utilizing primers prMD92 and prMD124, and the resulting PCR product was sequenced.

**Table 2:** PCR Primers.

Name	Sequence
prMND205.VexA.F	GATCA**CATATG**AAAAAAATCATCATATTACTAACGACATTTTTCC
prMND206.VexA.R	GATCA**GGATCC**TTAGAAAGAATTAGTGCCGCGGG
prMND214.Gad.F.XhoI	GATCA**CTCGAG**GCCGGAGGATCTGCCGTC
prMND215.Gad.R.KpnI	GATCA**GGTAC**CCCAAGCTTGCATGCCTGCAG
prMD92	GTTGCGGTAATGGTATAACGAAATAACAGATAC
prMD124	CACGCAATATTTCAATGATGGCAAC

Restriction sites are in bold.

### Stability of Ty21a-Gad

Ty21a-Gad was grown at high dilution in TSB-MES pH 5.5 with arabinose for ~ 24 hours, which represents ~25 generations of growth. This culture was serially diluted to ~10–100 cfu/ml and regrown each time for an additional 24 hours, for a total of 75 generations. Resulting colonies from agar platings, were individually grown and were analyzed by standard Western blotting procedures using the GadA/B specific antibody as below.

### Immunoblotting

Bacterial strains were grown as described above, normalized based upon culture OD, and the appropriate volume centrifuged. Pelleted bacteria were resuspended in lysis buffer (10 mM Tris-HCl pH 7.4, 10 mM MgCl_2_, 10 mM CaCl_2_ and 1% SDS), sonicated for 10 sec using a Microson ultrasonic cell disruptor, and mixed with LDS sample buffer (Life Technologies, NY). The samples were separated by Bis-Tris PAGE. Standard Western blotting procedures were carried out with anti-Gad A/B mouse polyclonal antibody [35] for identification of Gad A/B proteins.

### Intramacrophage survival assay

The U937 human monocyte cell line was obtained from ATCC. The intra-macrophage survival assay for *S*. Typhi was performed as previously described [36] with slight modifications. The cells were grown in RPMI 1640 (Gibco, NY) supplemented with 5% fetal bovine serum (FBS; Gibco) and 50 U/ml penicillin-streptomycin (Life technologies, NY) at 37°C with 5% CO_2_. Cells were centrifuged at 58 X g for 10 min, resuspended in fresh 37°C pre-warmed RPMI 1640 medium + FBS without antibiotics, adjusted to a concentration of ~1x10^6^ cells per ml, and 10 ml of cells were added to a 50 ml tube. After 24 hours, bacterial cultures were centrifuged and the bacterial pellets were resuspended in RPMI 1640 + FBS. The bacteria were added to U937 cells at a ratio of ~100 bacteria per U937 cell (time 0). After incubation at 37°C for 90 minutes, the infected U937 cells were centrifuged as before, washed 2 x with phosphate buffered saline (PBS) and resuspended in RPMI 1640 medium + FBS containing 250 μg/ ml of gentamicin in order to kill extracellular bacteria. After another 90 minutes (total time 3 hours), the cells were resuspended in 20 ml of fresh RPMI medium containing 25 μg/ml of gentamicin to prevent extracellular growth of any released bacteria. Additional PBS-washing of the cells was done at 24 hours post-infection. For viable bacterial count determinations at time 0, 3 and 24 hours, the infected macrophages were lysed with 0.1% Triton X-100 in PBS. After 3 min, cell lysates were serially diluted 10-fold in PBS, and aliquots were plated onto TSA plates to assess bacterial CFU. The bacterial CFUs were normalized by calculating bacteria per macrophage. All assays were repeated at least three times on different days. The results are presented as the mean ± SEM of all replicates. Statistical analyses were performed by means of an unpaired *t* test using GraphPad Prism version 5. A *p* value of <0.05 (two tailed) was considered to be statistically significant (**).

## Results

### Expressing Gad proteins in Ty21a

**Fig 2:**
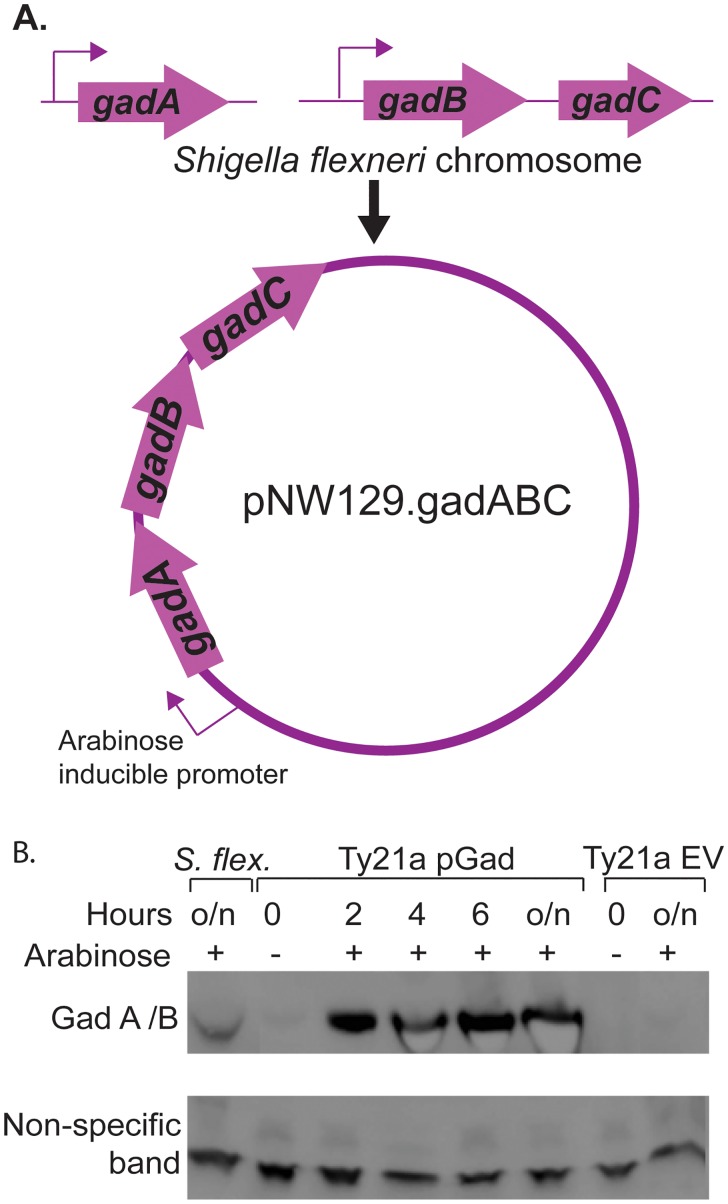
Expressing Gad proteins in Ty21a. **A.** The three *gad* genes, *gadA*, *gadB* and *gadC* were cloned in tandem with their own ribosome binding sites under the control of an arabinose inducible promoter in a multi-copy plasmid pNW129 [37] to express these genes free from the typical complex regulatory network. **B.** The expression of Gad proteins was determined by Western blot with anti-Gad A/B antibody [35]. Gad A and Gad B are 98% similar at the protein level. The cloned GadA/B are very weakly expressed in the absence of arabinose induction, but Gad A/B are expressed in significant amounts as early as 2 hours after arabinose induction and their expression is increased over time. Gad A/B are not expressed in the negative control Ty21a containing the empty plasmid vector (Ty21a EV), but are expressed in overnight cultures of *S*. *flexneri* strain CFS100. A non-specific, antibody-binding band was used as the loading control; qualitative, rather than quantitative, levels of GadA/B expression were observed under different conditions.

The Gad system of *Shigella* and *E*. *coli* is intricately regulated (Fig 1) [14, 35]. The genes *gadA* (S4173) and *gadBC* (S1867 and S1868) are found about 2000 kb apart on the *S*. *flexneri* 2a strain 2457T (AE016978.1 to AE016993.1) chromosome. These *gad* genes were cloned in series with their cognate ribosome binding sites under the control of an arabinose-inducible promoter (P_*ara*BAD_) in multi-copy plasmid pNW129 [37] to create pGad (Fig 2A). The intent was to make Gad proteins in response to added exogenous arabinose and, thus, free from the typical complex Gad-regulatory network. Since glucose is known to repress P_*ara*BAD_ [38], our initial studies used LB media, which does not contain glucose. However, for later studies we switched to TSB media, which contains 0.25% glucose, since better acid survival was seen following growth in TSB rather than LB (data not shown). We supplemented TSB media with 0.75% arabinose to counter any repression exerted by 0.25% glucose in standard TSB, and the arabinose inducer was present in all tested conditions, unless stated otherwise. The Gad proteins were detected by Western blot at the earliest examined time point of 2 hours after addition of arabinose to the Ty21a pGad culture. Gad protein expression appeared to increase over time (Fig 2B), suggesting that cloned Gad expression is essentially not sensitive to 0.25% glucose in TSB media in the presence of 0.75% arabinose.

### Extreme acid survival of Ty21a depends on growth phase

**Fig 3:**
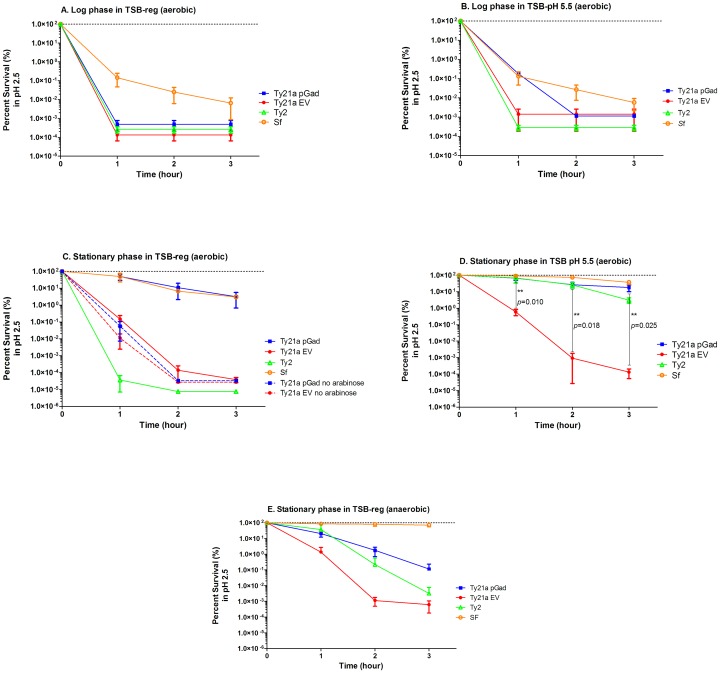
Extreme acid survival of bacteria depends on growth phase. **A and B**. When tested bacteria were pre-grown to log phase in commercial TSB or TSB-MES pH 5.5, under aerobic conditions, Ty21a with pGad (Ty21a pGad), Ty21a with empty vector (Ty21aEV), or wildtype *S*. Typhi Ty2 did not show any substantive survival at pH 2.5 after 3 hours. In contrast, *S*. *flexneri* (Sf) showed ~0.01% survival in media at pH 2.5 after 3 hours. **C.** However, when bacteria were grown to stationary phase (i.e. for 18 hours) in regular TSB under aerobic conditions, prior to pH 2.5 acid challenge, Ty21a pGad showed ~5 log increase in acid survival (i.e. comparable to survival of Sf), compared to the negative control Ty21a EV after 3 hours. Surprisingly, under these conditions Ty2 did not show any survival. Importantly, the ~5 log increase in acid survival of Ty21a pGad was dependent upon media supplementation with arabinose. D. When tested bacteria were pre-grown to stationary phase in TSB-MES pH 5.5 under aerobic conditions, prior to the pH 2.5 acid challenge, Ty21a pGad, Ty2 and Sf showed good acid survival, whereas Ty2la pEV demonstrated ~5 logs less acid survival than that of the other strains. The *p* value significance between Ty21apGad and Ty21a EV at 1, 2 and 3 hours are 0.010, 0.018 and 0.025, respectively. **E.** When tested bacteria were pre-grown to stationary phase in regular TSB under anaerobic conditions, prior to the pH 2.5 acid challenge, *S*. *flexneri* showed solid acid survival. However, the Ty21a strains and Ty2 showed poor acid survival.

To determine whether pGad can enhance the acid survival of Ty21a, selected bacterial strains were grown in regular TSB at pH7.3 or TSB-MES pH 5.5 until OD_600_ 0.4–0.5 (log phase, Fig 3A and 3B) or for 18 hours (stationary phase, Fig 3C and 3D). When Ty21a pGad was pre-grown to log phase in regular TSB, cells did not survive the subsequent pH 2.5 acid challenge even for 1 hour (Fig 3A). Note that, when grown in regular TSB, log-phase *S*. *flexneri* lost 4 logs of viability over 3 hours. By comparison, when Ty21a pGad was pre-grown to log phase in TSB-MES pH 5.5, viability at pH 2.5 for 1 hour was slightly improved, but bacteria succumbed to pH 2.5 after 2 hours (Fig 3B). Ty21a EV (empty vector pNW129 in Ty21a) and Ty2 did not show any substantive survival at pH 2.5 under either log phase pre-growth conditions. In contrast, *S*. *flexneri* showed low level survival (~ 0.01%) over 3 hrs. at pH 2.5 following these latter growth conditions. Overall, bacteria in log phase showed poor acid survivability.

In striking contrast, when grown to stationary phase in regular and/or mildly acidic TSB prior to pH 2.5 acid challenge, the acid survival of Ty21a pGad was enhanced significantly (~ 5 logs) compared to Ty21a EV. Although Ty21a EV in stationary phase was more acid resistant than that of log phase cells, most stationary phase Ty21a EV also succumbed after 2 hours at pH 2.5 (Fig 3C and 3D). The significant enhancement of acid resistance seen in Ty21a pGad was dependent, as expected, on the presence of arabinose (Fig 3C). In the absence of arabinose, Ty21 pGad acid survival was comparable to that of Ty21a EV. These data demonstrate that the *gad A*, *B*, and *C* genes cloned under the control of an arabinose-inducible promoter in the multi-copy plasmid pNW129 are responsible for significant enhancement of acid survival of Ty21 pGad. Although a substantial amount of Gad proteins are expressed in log phase Ty21a pGad (4 hours, Fig 2B), this strain in log-phase fails to withstand acid challenge (Fig 3A and 3B). In striking contrast, Gad proteins provide significant protection to stationary phase Ty21a against acid challenge (Fig 3C and 3D). These observations suggest that, in addition to Gad proteins, other ATR factors, expressed during stationary phase, contribute to optimal acid resistance. Surprisingly, Ty2 pre-grown in regular TBS at neutral pH for 18 hours, did not show any substantive survival under these conditions, whereas Ty21a pNW129 survived the acid challenge slightly better than Ty2 (Fig 3C). Most importantly, wildtype Ty2 pre-grown at mildly acidic pH 5.5 showed enhanced acid survival (~3% after 3 hours at pH 2.5; Fig 3D), most likely due to induced ATRs. The acid survival of *S*. *flexneri* pregrown to stationary phase was significantly enhanced relative to log phase, when pregrown either at pH 5.5 or 7.2; however, pre-growth under mildly acidic conditions stimulated maximal subsequent survival at pH 2.5. This is consistent with reports that in *Shigella* the native Gad system is not induced until late exponential phase [39] and full ATR are apparently expressed under subneutral pH growth conditions.

### Effect of aerobic vs. anaerobic growth on Ty21a acid susceptibility

A study by Hone et al., 1994 suggested that extreme acid survival of *S*. Typhi is optimum when the bacteria are pregrown in TSB media buffered at pH 5 under anaerobic conditions. Further, the arginine decarboxylase system used by *Salmonella* species to resist acid challenge is expressed only under anaerobic conditions [16, 40]. Thus, we investigated whether bacterial growth under anaerobic conditions prior to acid challenge could further enhance their acid resistance. We propagated the strains in regular TSB (pH 7.2), which contains 0.25% glucose, since anaerobic conditions caused the medium to fall below pH 5.5. The pH drop in the media, possibly due to glucose fermentation, has been observed by others [16]. Although *S*. *flexneri* showed solid acid resistance, neither Ty21a strains nor wild type Ty2 showed substantial acid resistance when propagated under anaerobic conditions (Fig 3E). Therefore, all remaining experiments were carried out under aerobic conditions.

### Effect of pre-growth media at pH ranging from 4.5 to 7.5 on extreme acid survival

**Fig 4:**
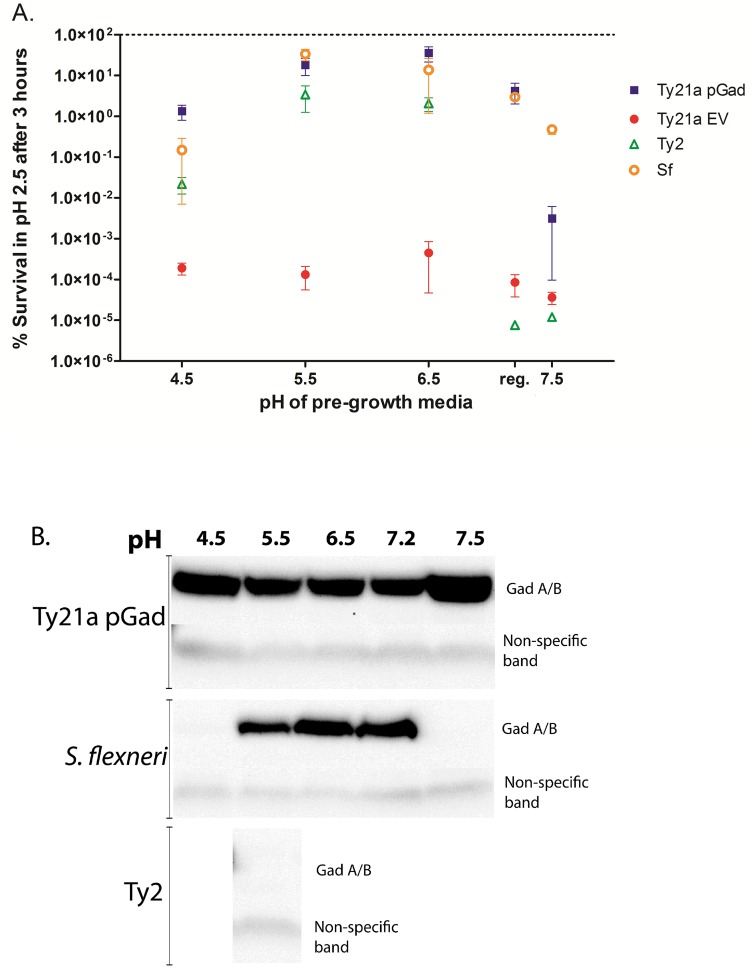
Extreme acid survival of *S*. Typhi strains depends upon the pH of the pre-growth media. **A.** Tested bacteria were pre-grown in either TSB-MES pH 4.5, TSB-MES pH 5.5, TSB-MOPS pH 6.5, commercial TSB (unbuffered pH 7.2), or TSB-MOPS pH 7.5 for 18 hours prior to the acid challenge, and survival at pH 2.5 for 3 hours was determined. Ty21a with pGad showed good survival when bacteria were pre-grown in mildly acidic pH media and in commercial unbuffered TSB, with the *p* value significance between Ty21apGad and Ty21a EV at pH 4.5, 5.5, 6.5 and 7.2 (unbuffered) are 0.0236, 0.0251, 0.0230 and 0.0468, respectively; however following pre-growth at pH 7.5, acid survival was decreased by ~ 3 logs (non-significant *p* value 0.1827). Our negative control Ty21a EV did not show any survival after pre-growth under all pH conditions. Although Ty2 showed good low pH-survival when pregrown at slightly acidic pH, it showed very poor survival when pre-grown at pH 7.5 or in commercial TSB at pH 7.2. Sf showed good survival under all pre-growth conditions, including pre-growth in TSB-MOS pH 7.5. **B.** The Gad protein expression of selected bacteria after 18 hours of growth in TSB-MES pH 4.5, TSB-MES pH 5.5, TSB-MOPS pH 6.5, unbuffered pH 7.2 TSB, and TSB-MOPS pH 7.5 was determined by Western blot with anti-Gad A/B antibody [35]. Gad protein is expressed under all the pH conditions in Ty21a pGad, where Gad protein expression is under the control of an arabinose inducible promoter. However, in Sf where Gad protein expression is tightly regulated, Gad protein expression is only seen in TSB-MES pH 5.5, TSB-MOPS pH 6.5 and unbuffered pH 7.2 TSB. As expected, the negative control Ty2 grown in TSB-MES pH 5.5 did not express any Gad proteins. An antibody-binding, non-specific band was used as a loading control; qualitative differences in GadA/B expression were monitored.

Next, we sought to study a range of pH of the pre-growth media for effect on extreme acid survival, possibly through induction of stationary phase ATRs. Bacteria were pre-grown in TSB-MES pH 4.5, TSB-MES pH 5.5, TSB-MOPS pH 6.5 or regular TSB (unbuffered, pH 7.2) or TSB-MOPS pH 7.5 for 18 hours, prior to the 3 hour acid challenge at pH 2.5 (Fig 4). Ty21a pGad demonstrated good survival when bacteria were pre-grown in mildly acidic pH media and regular unbuffered media. Although Gad proteins are expressed at pH 7.5 (Fig 4B), the acid survival of Ty21a pGad was about 3 logs lower at this growth pH. These data suggest that the ATR systems of Ty21a are not induced at pH 7.5; and, that the induction of ATR in addition to the Gad system is important for acid survival. Our negative control Ty21a pNW129, on the other hand, did not show substantive survival under any selected pH growth condition. Although Ty2 showed good acid survival when pre-grown at mildly acidic pH, it showed very poor survival when pre-grown at pH 7.5 or 7.2 (regular TSB), suggesting that the ATR of Ty2 is activated only at mildly acidic pH but not at neutral or slightly basic pH. These data further emphasize the fact that Ty21a acid survival mechanisms, such as inducible ATRs, cannot be optimally induced (or ATR mechanisms that are expressed are insufficient), to withstand extremely acidic conditions like the parent strain Ty2, likely due to mutations introduced by random mutagenesis during Ty21a construction. However, Ty21a pGad can achieve a remarkable level of acid resistance that is superior to Ty2 and comparable to Sf, but requires both the expression of Gad proteins and induction of ATR mechanisms under optimal growth conditions. *S*. *flexneri* exhibited substantial acid survivability under all pre-growth pH conditions including pre-growth in TSB-MOS pH 7.5. However, the *Shigella* systems necessary for maximal extreme acid survival were activated during pre-growth to stationary phase at pH 5.5.

### Effect of pH changes in unbuffered TSB cultures

It has been noted herein and previously that Ty21a is more acid sensitive than the parent strain Ty2. A previous study, in which Ty21a or Ty2 were pre-grown in mildly acidic TSB under anaerobic conditions for 18 hours before acid challenge, showed that the acid survival of Ty21a is greatly diminished compared to wild type strain Ty2 [22]. However, in the current study when the bacteria were pre-grown to stationary phase under mildly acidic aerobic conditions, Ty21a pGad showed 4 to 5 logs better acid survival than Ty21a EV (Fig 3D). Surprisingly, when selected bacteria were pre-grown in regular unbuffered TSB prior to acid challenge, Ty21a EV acid survival was ~3 logs superior to that of Ty2 after 1 hour (Fig 3C). Thus, the ATRs of Ty21a, but not Ty2, appear to be induced in regular TSB. When this anomaly was investigated further, we noticed that the pH of the Ty21a pGad and Ty21a EV cultures actually dropped below pH 6.5 after 18 hours of growth, whereas the pH of the Ty2 culture remained close to the initial pH 7.2. Thus, this observed pH decrease for Ty21a strains grown in regular TSB likely activates remaining ATRs, whereas similarly grown Ty2 do not activate ATRs because the pH remains at 7.2. However, pre-growth at pH 5.5 does optimally induce ATRs of Ty2, which make it more acid-resistant than similarly grown Ty21a EV (Fig 3D). Taken together, these observations demonstrate that the ATRs of both Ty2 and Ty21a are induced by growth at mildly acidic pH. Furthermore, under optimal ATR-inducing conditions, Ty2 is considerably more acid-resistant than Ty21a EV (Fig 3D).

### Glucose is important for induction of ATR

**Fig 5:**
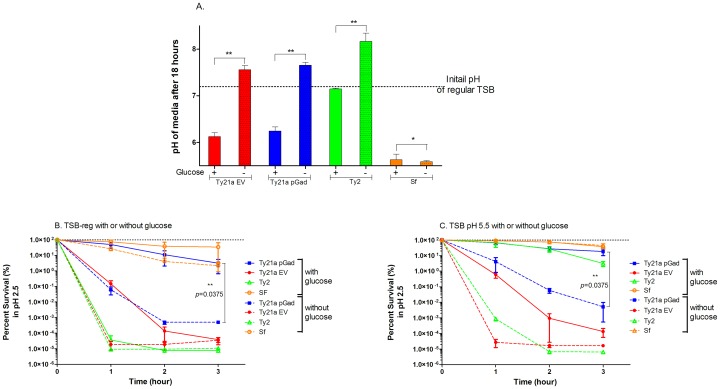
Glucose in the pre-growth media is important for extreme acid survival of *S*. Typhi strains. **A.** Bacteria were grown in commercial TSB supplemented with 0.25% glucose or TSB without added glucose for 18 hours and the pH of the cultures was determined. In the presence of glucose, the pH of Ty21a pGad and Ty21a pNW129 cultures dropped below 6.5 after 18 hours, whereas the pH of the Ty2 culture remained close to pH 7.2. The pH of the Sf culture dropped even further, to below pH 6. However, in the absence of glucose added to TSB, the pH drop was not observed with Ty21a strains, suggesting that glucose is responsible for the pH drop. The pH drop of Sf cultures was not dependent on glucose in the media. Data denoted by ** are significantly different (*p* < 0.05), but data marked * are not significant. **B.** Acid survival of bacteria was compared in the presence or absence of glucose in commercial TSB (pH 7.2). Acid survival ability of Ty21a pGad was greatly diminished (~ 3 logs after 3 hours) when the bacteria where grown in TSB without glucose. **C.** Acid survival of tested bacteria were compared in the presence and absence of glucose in TSB-MES pH 5.5. Acid survival ability of Ty21a pGad and Ty2 were significantly reduced (4 to 5 logs after 3 hours) when glucose was absent from the media. Even Ty21a acid survivability was reduced (~ 3 logs after 1 hour). However, Sf acid-survival was not affected by presence or absence of glucose in the media.

Under anaerobic conditions, glucose in the media is fermented by *S*. Typhi and the culture pH drops to ~5.5; however, under aerobic conditions, glucose is not fermented [16]. Therefore, we investigated whether glucose is responsible for the pH drop that was seen in Ty21a cultured in regular unbuffered TSB that contains 0.25% glucose. Selected bacteria were grown either in regular unbuffered TSB with glucose or TSB that does not contain glucose, for 18 hours and the pH of the cultures was determined (Fig 5A). In the absence of glucose in TSB, the pH drop was not observed with the cultured Ty21a strains, suggesting that glucose metabolism is responsible for the pH drop. Recently, *galE* mutations (such as that in Ty21a) have been reported to exert global defects on carbohydrate metabolism [41], which may explain the pH differences observed during growth in standard TSB between Ty2 and Ty21a. For *Shigella flexneri* cultured in standard TSB, the pH decreased at 18 hours to pH < 6 and this pH drop was not dependent on glucose in the media.

In order to determine the overall influence of glucose on acid survival, selected strains were grown in TSB with or without glucose prior to the pH 2.5 acid challenge. In TSB lacking glucose, Ty21a pGad survival at pH 2.5 was lower by ~ 4 logs after 3 hours and Ty21a EV survival was lower by 3 logs after 1 hour at pH 2.5 (Fig 5B), relative to strain grown in TSB with glucose. These data suggest that glucose metabolism in the pre-growth media of Ty21a strains is important for increased acid resistance by acidifying the media to activate the ATRs and/or provide an energy source for the activation of ATRs.

In order to decipher whether glucose metabolism simply acidifies the pre-growth media or plays a more direct role in activation of ATR, acid survival of selected bacteria were additionally compared in the presence and absence of glucose in TSB-MES pH 5.5 (Fig 5C). Acid survival of Ty21a pGad and Ty2 were significantly lower (4 to 5 logs after 3 hours) when glucose was absent from the media. Even glucose-deprived Ty21a EV acid survivability was reduced by > 4 logs after 1 hour at pH 2.5 when compared to glucose-grown Ty21a EV. These data suggest that glucose in the pre-growth media is important in direct activation of ATRs of Ty21a and Ty2, in addition to just acidifying the media of Ty21a cultures. In contrast, *S*. *flexneri* acid survival was largely unaffected by the presence of glucose in the pre-growth media. Nevertheless, *Shigella* acid-survival over 3 hours was increased slightly by ~1 log by pre-growth in glucose-containing standard TSB (Fig 5).

### Integration of *gad* genes into the Ty21a chromosome and removal of selectable antibiotic resistance

**Fig 6:**
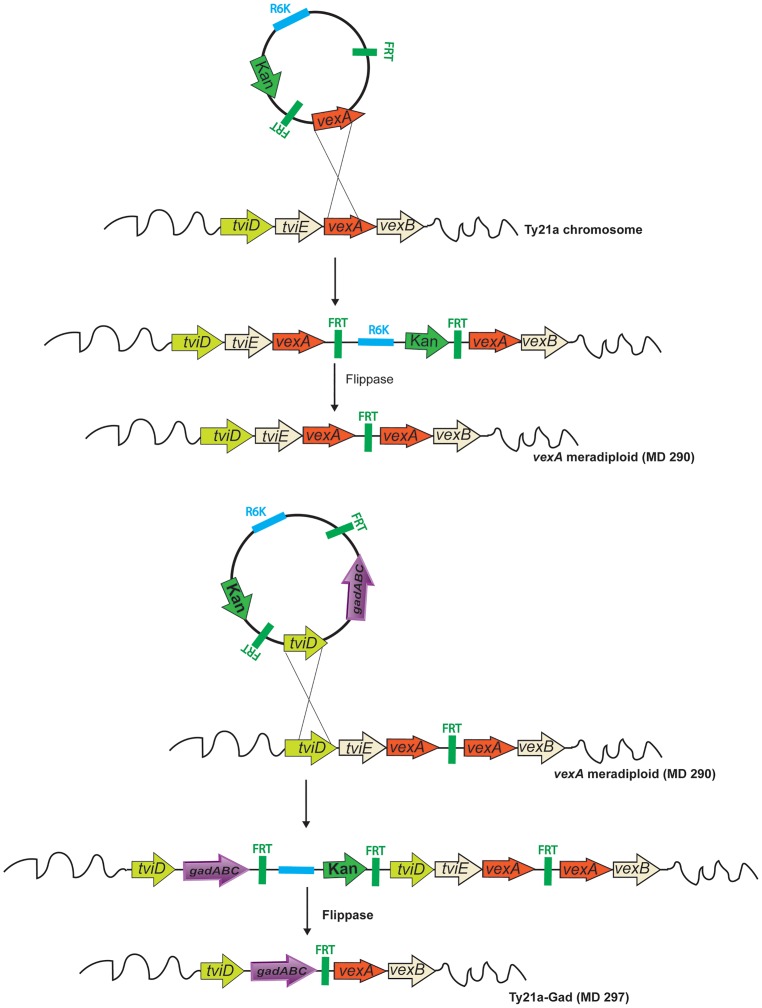
Integration of Gad genes into the Ty21a chromosome using a suicide vector. We constructed the R6K-based suicide vectors, pMD-SV and pMD-SD, that require a trans-supply of pir-encoded pi protein for replication. Although these plasmids can replicate in strains that express pi protein such as RHO3, efficient plasmid suicide results upon transfer to Ty21a which does not encode pir. Strain S17λpir carrying a Kan^R^ pMD-SV, which contains homology to the Vi operon *vexA* gene, was conjugated (utilizing RP4 as the conjugal transfer mediator) with Ty21a to transfer the pMD-SV plasmid. Kanamycin-resistant Ty21a with pMD-SV integrated into the Vi operon were first selected. Next, the region containing the Kan^R^ gene between the FRT (flippase recognition target) sites was eliminated by expression of the flippase enzyme from replication temperature-sensitive plasmid pCP20, resulting in the *vexA* merodiploid strain MD290. The *gad* genes under control of an arabinose promoter were cloned into pMD-SD (pMD-SD-gad), integrated into *vexA* merodiploid MD290, and the region between FRT sites was eliminated to construct the Kan^S^ Ty21a-Gad (MD297).

Since the Gad proteins must be stably expressed during vaccine manufacture and pGad contains an antibiotic resistance marker which is undesirable in manufacture of live human vaccines, we sought a technique to insert the *gad* genes into the Ty21a chromosome, that would allow for subsequent removal of the antibiotic selective marker. We have previously reported a modified λ red recombination method, termed super-recombineering, to insert large (10–20 kb regions) blocks of *S*. *sonnei* O-antigen genes into the chromosome of Ty21a [7]. We have subsequently used this technique to insert *S*. *dysenteriae* 1, *S*. *flexneri 2a*, and *S*. *flexneri 3a* O–antigen genes into the Ty21a chromosome (Dharmasena et al., unpublished manuscripts). Here, we describe a novel method to insert the *gad* genes into the Ty21a chromosome using a suicide vector and FRT sites that allow for the subsequent removal of the antibiotic resistance cassette and any unwanted plasmid regions with 100% efficiency. Most importantly, this new method utilizes the host homologous recombination pathway and does not require introduced λ Red enzymes. As schematically depicted in Fig 6, we constructed two plasmid R6K-based suicide vectors, pMD-SV and pMD-SD, that require a *trans* supply of the *pir*-encoded pi protein for replication. Although these plasmids can replicate in strains that express pi protein such as S17 λ*pir*, efficient plasmid suicide results upon transfer to Ty21a strains that lack *pir* [42]. The *asd* donor strain RHO3 containing pMD-SV, which contains sequences homologous to the Vi operon *vexA* gene, was conjugated with Ty21a to transfer the pMD-SV plasmid into Ty21a. Kanamycin-resistant Ty21a with pMD-SV integrated into Vi operon were selected and the region between the FRT sites was eliminated by expression of the flippase enzyme from plasmid pCP20 [34] to construct a *vexA* merodiploid strain MD290. Next, the *gad* genes, under control of an arabinose-inducible promoter, were cloned into pMD-SD (to generate pMD-SD-gad). Subsequent conjugal transfer of pMD-SD-gad into MD290 and recombination at *tviD* resulted in integration of the entire pMD-SD-gad. Subsequent introduction of flippase resulted in removal of the plasmid replicon and, *mob* sequences, Kan^R^ gene and one *tviD* gene, but left the introduced *gad* genes. This Ty21a-Gad chromosomal insert strain MD297 contains the *gad* genes integrated between single copies of *tviD* and *vexA* of the Ty21a Vi operon. As noted in Methods, these constructs were confirmed by PCR and DNA sequence analyses.

### Gad protein expression and acid survival of Ty21a-Gad

**Fig 7:**
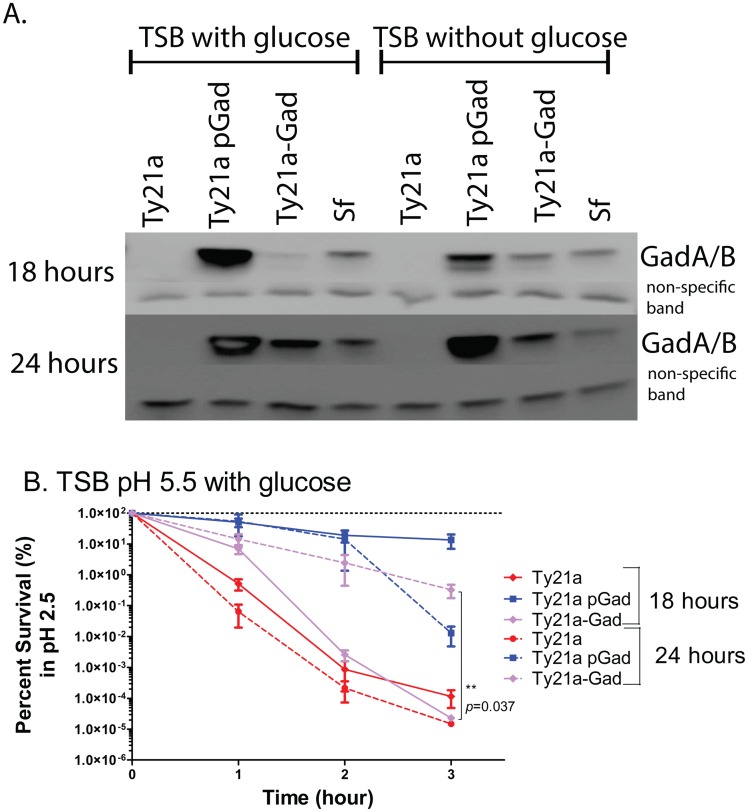
Chromosomal integrant (Ty21a-Gad) can survive extreme pH when pre-grown for 24 hours under appropriate inducing conditions. **A.** Selected bacteria were grown in TSB-MES pH 5.5 with or without glucose for 18 or 24 hours prior to acid challenge. Although Ty21a-Gad did not express much detectable Gad protein at 18 hours in the presence of glucose, Gad-expression was easily observed at 24 hours. Interestingly, Gad proteins were expressed even in the absence of glucose after 18 hours. In contrast to single copy Ty21a-Gad, the plasmid construct (Ty21a pGad) existed at ~ 10 copies per cell [44] and expressed a higher level of Gad proteins under all conditions. An antibody-binding, non-specific band was used as the loading control. **B**. Consistent with level of Gad protein expression, Ty21a-Gad acid survival was comparable to that of Ty21a after 18 hours of growth in TSB-MES pH 5.5 with glucose, but acid survival improved significantly (~3 logs) when Ty21a-Gad were grown for 24 hours prior to acid challenge.

Next, Gad protein expression of the chromosomal integrant (Ty21a-Gad) was compared to that of the plasmid construct (Ty21a pGad). Bacteria were grown in TSB-MES pH 5.5 with or without glucose for 18 or 24 hours prior to acid challenge. The arabinose-inducible promoter, P_*ara*BAD,_ that controls Gad protein expression here, is typically repressed by glucose. However, Gad protein expression from the multi-copy plasmid construct was not repressed in standard TSB-glucose containing 0.75% arabinose after 2, 4, 6(Fig 2), 18 or 24 hours (Fig 7), and expressed a high level of Gad proteins even in the presence of glucose. In contrast, the single copy chromosomal integrant, which is also under the control of P_*ara*BAD_, was considerably more sensitive to glucose repression in standard TSB and did not show much Gad protein expression at 18 hours in the presence of glucose. However, a moderate amount of Gad protein expression was seen by 24 hours growth (Fig 7A). Since *Salmonella* catabolize glucose as its main energy source [43], it is likely that the glucose in the media is gradually depleted and Gad protein expression is increased over time. Note that even in the absence of glucose in the TSB, after 18 hours, Gad proteins were expressed only moderately (but in similar amounts to the *Shigella* positive control) from the chromosomal integrant. Overall, Gad protein expression from the chromosome was reduced relative to that observed from the multi-copy plasmid with ~ 10 copies/cell [44]. However, since glucose is essential for optimal ATR expression in Ty21a, growth for 24 hours allowed for both Gad protein expression and ATR induction. Furthermore, no Gad protein expression was detected when Ty21a-Gad was grown in the absence of arabinose (data not shown) suggesting that *gadABC* integrated into the Vi operon is exclusively regulated by *araC* promoter.

We next compared the acid resistance afforded Ty21a by the Gad chromosomal integrant versus plasmid construct after growth in TSB-MES pH 5.5 for 18 and 24 hours. Consistent with the observed level of Gad protein expression after 18 hours, Ty21a-Gad showed poor acid survival and was comparable to that of theTy21a control at 18 or 24 hours. However, acid survival improved significantly (~3 logs) when Ty21a-Gad were grown for 24 hours prior to acid challenge (Fig 7B). Under these conditions, Ty21a pGad grown for 18 hours showed the best acid survival over 3 hours. Thus, these data suggest that Ty21a acid survival depends upon both ATRs and the level of Gad protein expression. Finally, Ty21a pGad and Ty21a-Gad grown in TSB in the presence or absence of arabinose showed identical growth curves to that of Ty21a alone (data not shown), suggesting that Gad protein expression does not affect the basic growth characteristics of Ty21a.

Stability of recombinant Ty21a-Gad, which is antibiotic-sensitive and contains chromosomally integrated *gad* genes under the control of arabinose inducible promoter was tested by immunoblotting after ~75 generations of growth to assess genetic stability. The resulting cells after 75 generation were plated and 60 colonies were individually grown to test for GadA/B expression as described in Materials and methods. All of 60 colonies tested retained Gad A/B expression (data not shown), demonstrating 100% stability of the chromosomally integrated genes.

### In vitro intramacrophage survival

**Fig 8:**
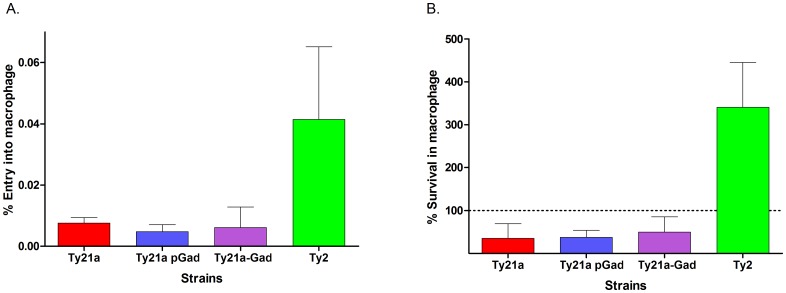
Gad-induced acid resistance in Ty21a does not alter strain attenuation, as determined by entry into macrophages (A) and survival in macrophages (B). Entry into macrophages and survival in macrophages are key virulence traits of wildtype *S*. Typhi that have been lost due to mutation in Ty21a [36]. Macrophage entry and survival of strains expressing Gad proteins (Ty21a pGad and Ty21a-Gad), Ty21a and Ty2 were performed as previously described [36]. A. The percent of bacterial entry was calculated as the ratio of bacteria that entered into cultured human U937 macrophages, 3 hours after infection compared to the initial bacterial inoculum. Ty21a pGad (0.005%) and Ty21a-Gad (0.006%) showed low entry, comparable to Ty21a (0.008%), whereas Ty2 showed about ~5 fold better entry into macrophages (0.04%). B. The percent survival was calculated as the percentage of bacteria inside cultured human U937 macrophage cells 24 hours after infection, compared to bacteria internalized in macrophages at 4 hours. Ty21a pGad (37%) and Ty21a-Gad (49%) showed low survival, comparable to Ty21a (35%), whereas Ty2 showed robust replication within macrophages (341%). The percent survival of Ty21a pGad, Ty21a-Gad and Ty21a was significantly lower than that of Ty2 (*p* values were 0.0077, 0.0104and 0.0086 respectively; see Methods for statistical analyses).

*S*. Typhi is a human-specific pathogen that does not naturally infect any animal species including monkeys. Typhoid fever is a disease of the reticuloendothelial system and the ability to survive and replicate within macrophages/monocytes is thought to be one of the major pathogenic determinants traits of *S*. Typhi, which helps them disseminate via the systemic circulation to other sites of infection [45]. *S*. Typhi survive and replicate within macrophages by adapting to conditions within fused phagolysosomes, which include low pH, as they do not inhibit phagosome-lysosome fusion [46]. Since there is no natural disease animal model for this organism and a key pathogenic feature of *S*. Typhi involves its ability to survive in macrophages, cultured human macrophage cell lines have been used as an surrogate model to analyze relative attenuation [36]. Previously, when Ty21a was compared to its virulent parent Ty2 using the human monocyte-macrophage cell line U937, Ty21a showed markedly reduced entry and very low intra-macrophage survival, whereas Ty2 entered macrophages at a higher rate and exhibited 100% survival and robust replication within U937 cells [36]. Thus, we determined the entry and intra-macrophage survival of Ty21a, Ty21a pGad and Ty21a-Gad in comparison with Ty2, using the human monocyte-macrophage cell line U937. As expected from previous studies [36], Ty21a, Ty21a pGad and Ty21a-Gad showed very low macrophage entry and survival compared to Ty2 and were not observed to replicate within macrophage cells. In contrast, Ty2 showed efficient entry and intra-macrophage survival and robust replication within these macrophages over 24 hours (Fig 8A and 8B). These data demonstrate that addition of the Gad genes to strains Ty21a pGad orTy21a-Gad did not alter the previously reported attenuation of Ty21a for internalization into macrophage or for intra-macrophage survival.

## Discussion

In this study, acid survival of Ty21a was greatly enhanced by the combined expression of *Shigella* Gad AR proteins and bacterial growth under ATR-inducing conditions. For example, when Ty21a were grown to stationary phase in mildly acidic media containing glucose, the ATR was optimally induced, but cell count decreased 6 logs at pH 2 over 3 hours. In contrast, when Gad proteins were expressed from an arabinose-inducible promoter under optimal ATR-inducing conditions, Ty21 pGad acid survival was enhanced by ~5 logs over a 3 hour period at pH 2.5, and survival equaled that seen with the highly acid-resistant *Shigella flexneri* control (Fig 3D). Under these optimal conditions, the parent Ty2 strain was significantly more acid-tolerant than Ty21a alone. However, the acid survival of strain Ty2 was strictly dependent on optimal ATR inducing conditions and absence of any one of several required conditions resulted in a 4–5 log reduction in acid survival. These results suggest that the infectious dose of *S*. Typhi may vary greatly depending on nutritional sources and environmental conditions. For example, the infectious dose of *S*. Typhi is thought to range from ~10^5^ to 10^6^ cfu based upon volunteer challenge studies in which *S*. Typhi was administered in skim-milk [47], but epidemiological studies suggest a lower infectious dose of 10^3^ cfu during water-borne outbreaks of *S*. Typhi [25].

RpoS plays an important role in triggering general stress responses to protect bacteria against environmental challenges that include nutrient starvation, variations in temperature, osmolarity, or pH [48]. In *S*. Typhimurium, two distinct ATR systems are expressed in stationary phase. One system is dependent on RpoS and the other is RpoS-independent and acid-inducible. The ASPs induced during the log phase ATR are regulated by RpoS, PhoP/Q and Fur. However, the stationary phase ATR provides more sustained and better protection than the log phase ATR [19]. Our results demonstrate that Ty2 and Ty21a cells in stationary phase are more acid-resistant than those in log phase. Since Ty2 and Ty21a strains express an elongated, suboptimal RpoS due to a frameshift mutation [49], the major acid survival mechanism that is active under aerobic conditions is the acid-inducible, RpoS-independent ATR. Ty21a shows poor acid survival even under our defined ATR-optimal growth conditions and may not be able to express all the ASPs that contribute to the RpoS-independent ATR due to yet uncharacterized mutations introduced by random mutagenesis during development of Ty21a [23]. Nevertheless, acid survival of Ty21a at pH 2.5 for 3 hours is enhanced by an impressive 5 logs with the combined expression of Gad proteins plus induction of the remaining ATR mechanisms.

Since Ty21a pGad showed better acid survival under aerobic conditions, which are more desirable for vaccine manufacture, we studied acid-resistance more thoroughly under aerobic conditions. The acid survival of *Salmonella*, mediated by the native arginine (AR3), lysine (AR4), or ornithine (AR4) decarboxylases, is strictly dependent on anaerobic conditions and these decarboxylases are not typically expressed under aerobic conditions [16, 24]. Recently, Brennerman et al. 2013 cloned the AR3 arginine decarboxylase system under rhamnose-inducible promoters and increased the acid-resistance of *Salmonella* recipient strains grown under aerobic conditions. However, survival of these acid-improved strains was still reduced 5 logs after 3 hours at pH 2.5 and did not approach the impressive, Gad-enhanced and sustained, high acid-resistance levels reported herein.

The Gad system that we used in this study consists of cytoplasmic, paralogous GadA and GadB decarboxylases and an inner membrane bound antiporter, GadC. Gad A and B form 330 kDa hexamers assembled from trimerization of GadA(B) dimers. When the bacterial cells are exposed to extreme pH (~2.5), the cytoplasmic pH drops to ~ pH 4.5 and the N-terminal domain of these two isozymes undergoes a conformational change that allows interaction with the inner membrane to form the active form. The active enzymes convert glutamate to GABA and CO_2_ in a reaction that consumes a cytoplasmic proton. The inner membrane antiporter GadC transports GABA out of the cell in exchange for more glutamate (Fig 1). However, when the cytoplasmic pH returns to neutral, GadAB is returned to an inactive form [14]. Therefore, Gad proteins should be optimally expressed in Ty21a during vaccine manufacture such that when the bacteria encounter extreme acidic conditions in the stomach, bacteria are ready to respond to the acid stress rapidly without the need for protein synthesis. If this vaccine strain is delivered orally as a dried wafer, Gad function will only be activated when the cells are exposed to low pH. Most importantly, our in vitro studies have clearly shown that Gad-expression in Ty21a significantly improves extreme acid (pH 2.5) survival over a prolonged human gastric transit time of 3 hours at pH 2.5, mimicking transit through a full stomach. Even though it produced a marked improvement in acid resistance of Ty21a, Gad expression did not modify predictable strain attenuation as assessed by uptake and survival within human macrophages or normal growth patterns. Intra-macrophage/monocyte survival is a key virulence attribute of *S*. Typhi. Moreover, Ty21a has never been detected in peripheral blood monocytes of volunteers at any time point following oral administration [50] suggesting that a key attenuation mechanism for Ty21a affects uptake into and survival within macrophages. From the above studies, we believe that the precise recombinational events that were used to create the acid-resistance modification of Ty21a did not alter the immunogenicity or attenuation of this oral bacterial vector.

*S*. Typhi is a human specific pathogen, and there are no validated animal models that can be used to assess the effects of gastric acidity or oral immunogenicity of candidate oral, attenuated *S*. Typhi vaccines. Thus, animal models cannot, at present, provide data that are relevant to human responses. Instead, human clinical studies will likely be required to demonstrate that the improvements in Ty21a acid-resistance actually allow for improved gastric passage and enhanced overall immune responses relative to unimproved Ty21a, analyses that are beyond the scope of the current study.

## Conclusions

The acid resistant Ty21a candidate vaccine strains were pregrown aerobically in mildly acidic TSB media, which contains glucose, until the cells reach stationary phase for optimal acid survival. Thus, manufacture of an acid-resistant Ty21a vaccine candidate will likely require recapitulation of these conditions. Acid-resistant Ty21a delivered in a rapidly dissolvable wafer format to the sublingual lymphoid tissue and the entire lower gastrointestinal tract could comprise a superior delivery system for foreign antigens from numerous infectious agents. This format may ultimately enhance specific immune responses, possibly allowing for a reduction in dose number, and would be more child-friendly. Moreover, this acid-resistance pathway, which functions in the cytoplasm of Ty21a expressing *gad* genes, should not interfere with normal gastric physiology. Further, this acid-resistance system may be extendable to other live oral vaccine strains (e.g. attenuated *Salmonella spp*., *Vibrio cholerae*) to improve oral delivery formats.
